# Giant Hepatic Hemolymphangioma With Peritoneal Effusion in Children: A Case Report and Literature Review

**DOI:** 10.3389/fped.2022.817521

**Published:** 2022-02-18

**Authors:** Yufeng Li, Lei Ta, Yuan Xu, Jianli Liu

**Affiliations:** ^1^Department of Radiology, Lanzhou University Second Hospital, Lanzhou, China; ^2^Second Clinical School, Lanzhou University, Lanzhou, China; ^3^Key Laboratory of Medical Imaging of Gansu Province, Lanzhou University Second Hospital, Lanzhou, China

**Keywords:** child, liver, hemolymphangioma, peritoneal effusion, case report

## Abstract

Hemolymphangioma is a congenital malformation of blood vessels and lymphatic vessels, commonly found in the head, neck, and subcutaneous, rarely in the viscera and extremely rarely in the liver. In this case, a 6-year-old boy was found to have abdominal distension for more than 2 months with no other obvious symptoms. Physical examination revealed a large abdominal mass that was hard and not mobile. Laboratory tests found no obvious abnormity. Preoperative ultrasound and CT showed a huge cystic and solid-cystic tumor in the abdomen with close relationship to the right lower margin of the liver and fluid accumulation in the abdominopelvic cavity. The preliminary diagnoses were a malignant tumor of embryonic origin and undifferentiated sarcoma. Liver tumor resection was performed in our hospital, and the postoperative pathology was diagnosed as hepatic hemolymphangioma. The patient recovered well after surgery. It is easy to diagnose a large abdominal mass in a child as a malignant tumor of the liver and delay the treatment—no obvious symptoms, no obvious abnormalities in laboratory tests, and imaging shows a multiocular cystic lesion with clear borders and no invasion of blood vessels, indicating that the possibility of this disease should be considered. The tumor has an abnormal rich blood supply, and preoperative imaging evaluation clearly shows the vascular pathway and blood supply status to help optimize the surgical plan.

## Introduction

Hemolymphangioma is a congenital malformation of blood vessels and lymphatic vessels, which is a non-genuine tumor. Its occurrence in the liver is extremely rare, and only three cases (1–3) have been reported in the literature by far (PubMed, Medline, Chinese Biomedicine Database and the China Journal Full Text Database), of which only two original articles of adult cases are available (2, 3). In this paper, a case of huge hepatic hemolymphangioma in a child is reported and analyzed in the context of previous literature reports.

## Case Presentation

### Case History

The child was 6 years old, male, with abdominal distension found 2 months before admission, with no obvious precipitating factors and no abdominal pain or diarrhea. A large abdominal mass was palpated on physical examination, hard and without obvious mobility; the child's development was normal. Laboratory tests (including blood routine, CRP, liver function, bilirubin, AFP, CA125, etc.) did not show any significant abnormalities.

### Imaging Studies

Ultrasonography showed a huge multiocular cystic mass in the right lower abdomen with regular morphology, intact envelope, close relationship with the liver, uneven internal echogenicity, scattered anechoic areas within the hyperechoic zone, multiple hyperechoic light bands within the cyst wall, and abundant blood flow signal within the mass, with no obvious dilatation of portal vein and intrahepatic bile duct.

**Figure 1:**
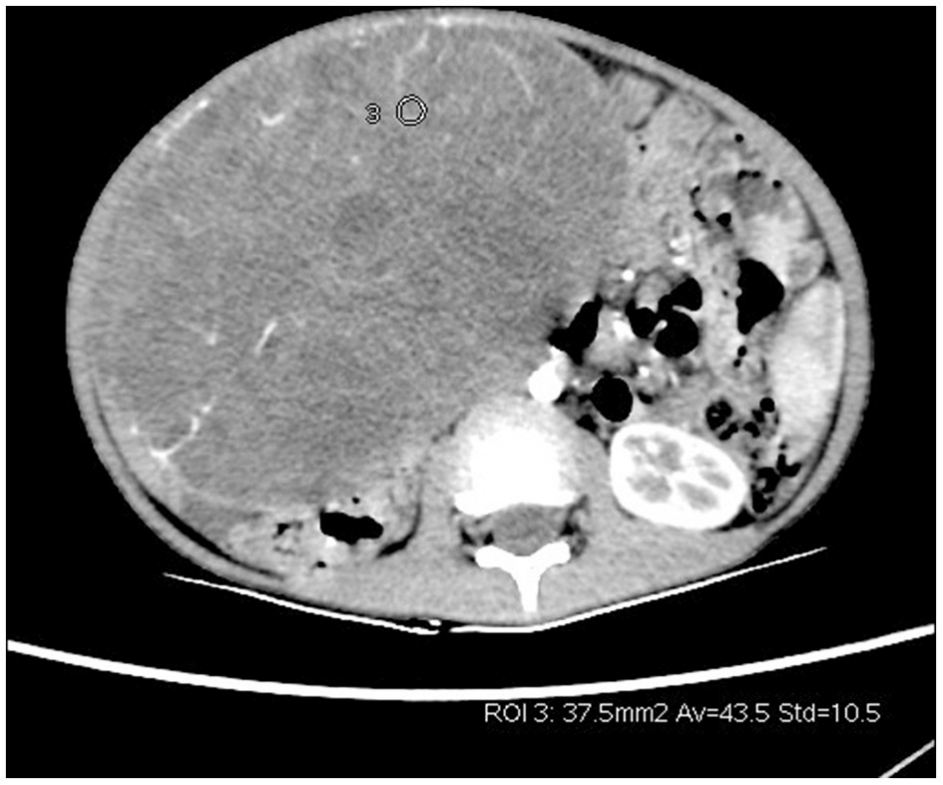
Enhanced CT of the arterial phase of a giant solid-cystic lesion in the right lower abdomen with abundant tumor vascularity.

**Figure 2:**
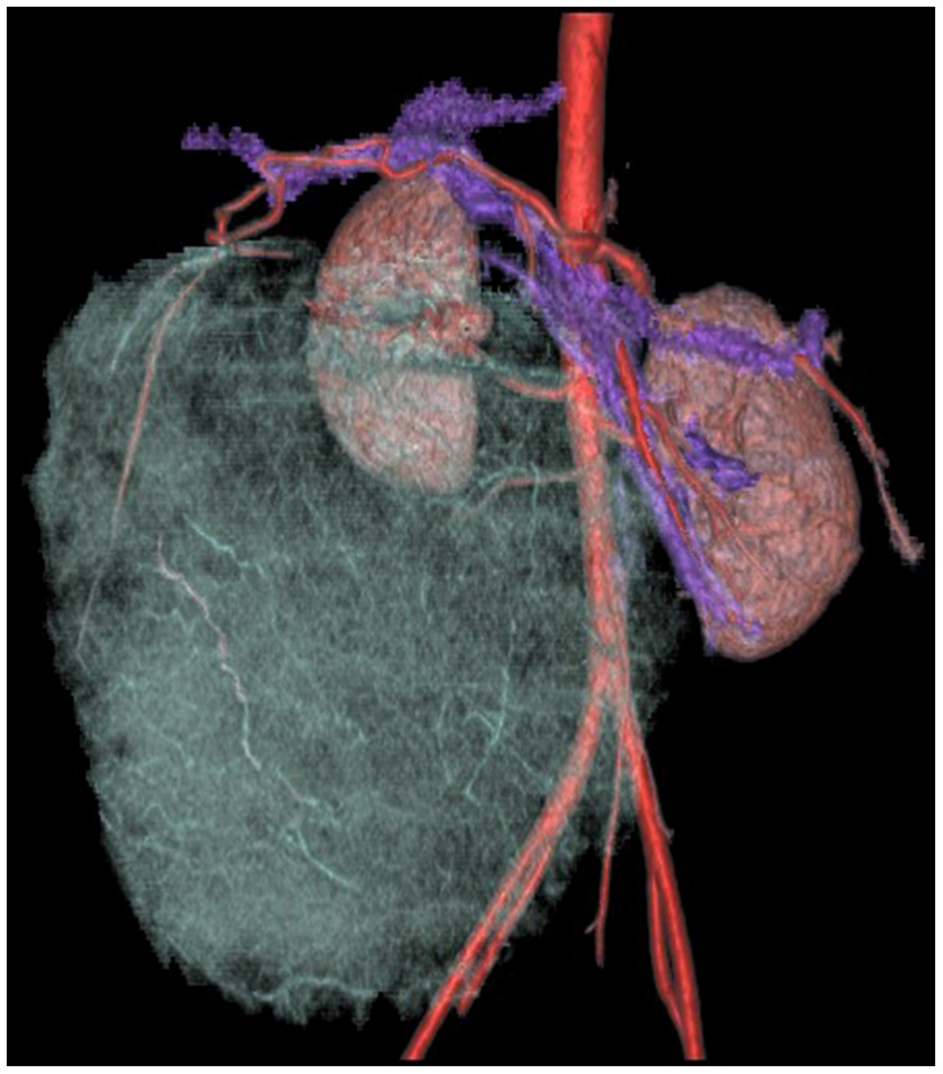
Enhanced CT of progressive enhancement of the solid part of the lesion in the portal venous phase, the lesion closely related to the liver, and the abdominopelvic fluid accumulation.

**Figure 3:**
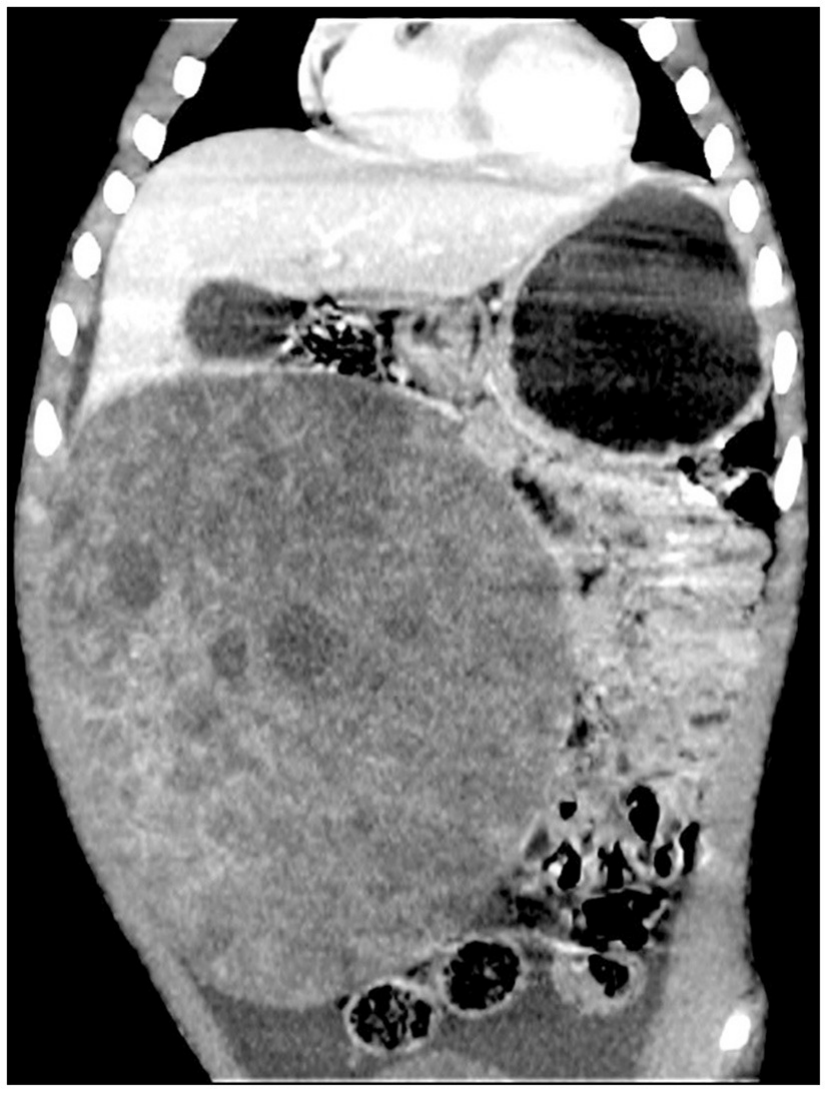
Enhanced CT of three-dimensional reconstruction. The adjacent right branch of the proper hepatic artery, abdominal aorta, right common iliac artery, and mesenteric vein was displaced by compression, and the distal branches of the hepatic artery were penetrated within the tumor.

CT scan + enhancement of abdomen showed a huge mixed-density mass in the right lower abdomen with clear borders and multilocular cystic solidity, about 11 × 16 × 17 cm in size; enhanced scan showed heterogeneous enhancement, in which the solid component and cystic wall and separation showed progressive enhancement, with CT values of about 44, 52, 65, and 77 HU in the plain, arterial, portal venous, and delayed phases, respectively (Figures 1, **3**). No significant enhancement was seen in the cystic component; the mass was closely related to the right lower margin of the liver which showed a “cup-like” change, and branches of the intrinsic hepatic artery were seen in the arterial phase penetrating the mass (Figures 2, 3). The adjacent organs were displaced by pressure; fluid accumulation was seen in the abdominopelvic cavity, and no enlarged lymph nodes were seen (Figure 3); the preliminary diagnosis of the radiologist was (1) embryonic malignant tumor of liver origin, and (2) undifferentiated sarcoma of liver.

### Treatment and Pathological Manifestations

The tumor was huge, rich in blood vessels, and accompanied with ascites, so we thought it was a malignant tumor before operation. Due to the abnormally rich blood supply of the tumor, there was a certain risk in puncture biopsy, and tru-cut biopsy was not performed before operation. In addition, because the tumor boundary was clear, after multidisciplinary discussion, we decided to carry out exploratory laparotomy, resection of liver segment V tumor, and abdominal drainage. Exploratory laparotomy revealed yellow fluid outflow and a large grayish brown mass emanating from the right margin of the liver. The adjacent blood vessels of the tumor were ligated, the mass was separated by a microwave knife, and complete resection of the tumor and a small amount of normal liver tissue of segment V performed. After the operation, the child recovered well and the incision healed; the blood routine showed hemoglobin (117 g/L, normal range 118–156) and the average red blood cell volume (76.3 fl, normal range 77–92) was slightly reduced. Biochemical parameters showed that alanine aminotransferase (84 U/L, normal range 7–30) and γ-glutamyl transpeptidase (35 U/L, normal range 5–19) increased. It is recommended that patients be reviewed regularly after 1, 3, and 6 months later.

#### Pathological Gross Observation

**Figure 4:**
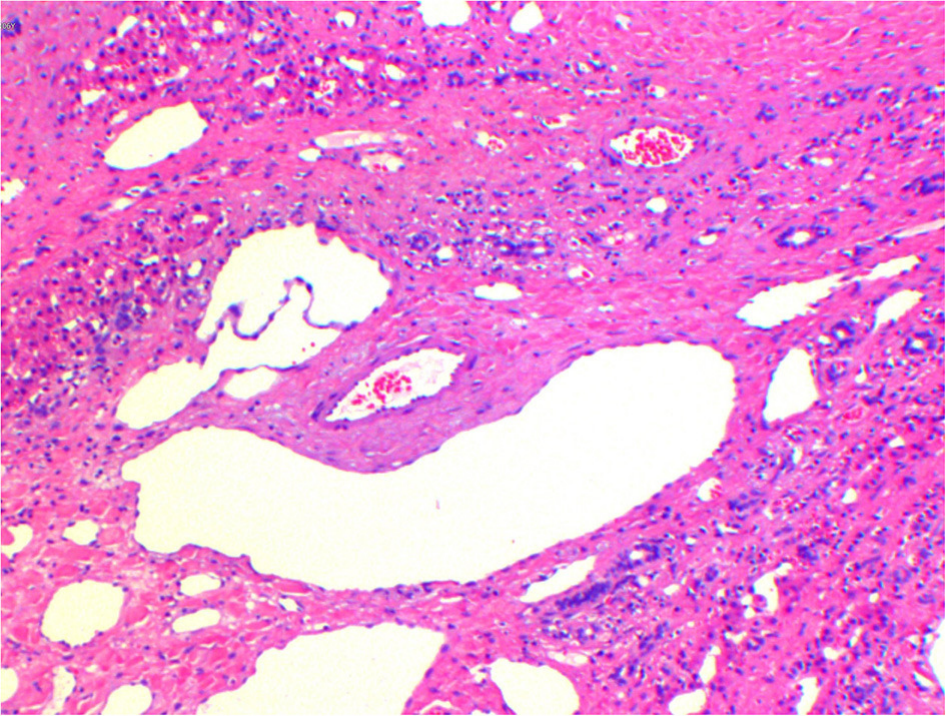
Pathological HE staining. The tumor consists of abnormally dilated multicystic-like lymphatic vessels and blood vessels, in which hepatocytes and small bile ducts were seen.

The mass was cystic solid, about 10 × 15 × 16 cm, with intact peritoneum and regular borders; the cut surface was grayish white, grayish brown, medium in texture, locally multilocular cystic, containing yellowish green clear fluid. Microscopically, the tumor was seen to consist of abnormally dilated multilocular lymphatic vessels and blood vessels; hepatocytes and small bile ducts were seen inside (Figure 4). Immunohistochemistry: vascular endothelial D2-40 cells (+), CD34 (+), CD31 (+), cell proliferation antigen Ki67 (3% +). Pathological diagnosis: hepatic hemolymphangioma.

### Outcome and Follow-Up

Ultrasound review at 3 weeks postoperatively showed that the operation area recovered well and no tumor residue was found. There was no abnormality in the physical examination and laboratory examination (CRP, blood routine, and biochemical parameters). After 3 months of follow-up, the child has good growth and development, no abnormal discomfort symptoms, and no abnormality in ultrasound and laboratory examination.

## Discussion

Hemolymphangioma is a congenital malformation of the vascular and lymphatic system, the formation of which may result from obstruction of the venolymphatic communication between the dysembryoplastic vascular tissue and systemic circulation (4, 5).

It is commonly found in loose connective tissues such as the head, neck, subcutaneous, etc. Its occurrence in the liver is extremely rare, and after searching (PubMed, Medline, Chinese Biomedicine Database, and the China Journal Full Text Database), as of October 10, 2021, there were only three reports on hepatic hemolymphangioma (1–3). Among them, Daudet (1) first reported a case of hepatic hemolymphangioma in an infant in 1965 and the original article could not be accessed. We referred to the other two publications to provide a comparative analysis of the clinical and imaging features of hepatic hemolymphangioma.

The clinical symptoms of abdominal hemolymphangioma are atypical, often presented with abdominal distension, abdominal pain, or palpable abdominal masses (6–9), and most cases show no significant abnormalities in laboratory tests; some cases are reported to be complicated by anemia (6–9), and those occurring in the gastrointestinal tract often come with secondary hemorrhage (9, 10). In the present case, the child presented with a right lower abdominal distension, with no specific clinical signs and symptoms and no significant abnormalities in laboratory tests. Abdominal hemolymphangioma can occur at all ages, and the two available cases of hepatic hemolymphangioma were 52 and 26 years old, respectively (2, 3). Imaging of hemolymphangioma is mostly a well-defined multilocular cystic or solid-cystic mass with variable cavity size and thin cystic wall. The amount of solid component is related to the blood vessel and lymphatic vessel content (5), and multiple small cysts are usually seen within the solid portion. The cystic component may be the result of ruptured and fused lymphatic vessels, and the solid component represents a remnant and compressed vascular tissue (4–6). In the present case, the mass was a multilocular cystic solidity with uneven wall thickness and cystic cavities of varying sizes within the solid component. The enhancement scan shows generally heterogeneous density, with multiple small internal arterial penetrations and mild progressive strengthening of the cyst wall, compartments, and solid parts, with more pronounced strengthening in the delayed phase, presumably due to the high vascular content and predominantly venous nature of the mass. Zhang et al. (3) reported a case with multiple cystic cavities within the solid component with a honeycomb shape, and the imaging presentation was similar to the present case. In addition, the imaging of this case showed fluid in the abdominopelvic cavity, which may be due to tumor compression and obstruction of blood return in combination.

As a benign tumor, hemolymphangioma is treated by surgical resection, which has a good prognosis. However, its potential aggressiveness has been confirmed in the literature (11–13), and postoperative recurrence is occasionally seen, requiring periodic review. In this case, a routine resection of the liver tumor was performed. On exploratory laparotomy, a yellowish fluid flow was seen, along with a large grayish-brown mass emanating from the right margin of the liver, and the remaining abdominal organs showed no significant abnormalities, so complete resection of the mass and a small amount of normal liver tissue was performed. No tumor residue was seen on ultrasound review 3 weeks after surgery, and the child recovered well on physical examination.

A large abdominal mass with abdominopelvic fluid is easy to be misdiagnosed as hepatoblastoma, undifferentiated embryonal sarcoma of the liver, and infantile hemangioendothelioma of the liver. Hepatoblastoma is usually located in the right lobe of the liver, grows exophytically, and usually presents as a large round-like or irregular lobulated mass with pseudo-envelope and well-defined borders; CT scan is mixed isointense or hypointense, which is always less dense than normal liver tissue, with internal hemorrhage, necrosis, cystic changes, and calcification. Enhancement scan is heterogeneous (14). Undifferentiated embryonal sarcoma of the liver is mostly located in the right lobe of the liver, and the early stage is confined to the swelling growth of the liver with a pseudo-envelope, and the boundary with the surrounding tissues is clear. CT plain scan shows a huge cystic or solid-cystic hypodense mass with well-defined borders; internal hemorrhage, necrosis, and cystic lesions are visible, and the proportion of cystic component is satisfactory, with the solid component mostly located at the edge. Enhancement scan shows a mild patchy enhancement of the solid part at the edge, the internal separation shows continuous progressive enhancement, and thickened tortuous arterioles are seen in the compartment (15, 16). Infantile hemolymphangioma of the liver is commonly seen in infants under 6 months of age as a cystic, cystic-solid mass, which appears on CT as a well-defined hypodense mass shadow in the liver parenchyma with mostly scattered central calcifications in granular aggregates (17).

## Conclusion

Hemolymphangioma is extremely rare in the liver. It has no specific symptoms and no obvious abnormalities in laboratory tests, and the tumor is usually large. When it occurs in children, it can easily be misdiagnosed as a malignant tumor. If imaging shows a huge multilocular cystic lesion with mild progressive enhancement of the cyst wall, separation, and a solid portion, with clear borders, this needs to be considered a possibility of hemolymphangioma. As a benign tumor, hemolymphangioma is treated by surgical resection, which has a good prognosis. However, this tumor has an abnormally rich blood supply, and the adjacent large vessels are obviously compressed. Preoperative imaging evaluation clearly shows the vascular alignment and blood supply condition, which can help optimize the surgical plan.

## Data Availability Statement

The original contributions presented in the study are included in the article/Supplementary Material, further inquiries can be directed to the corresponding author.

## Ethics Statement

The present study was approved by the Ethics Committee of Lanzhou University Second Hospital (approval number: 2021A-564). Written informed consent to participate in this study was provided by the participants' legal guardian/next of kin. Written informed consent was obtained from the individual(s), and minor(s)' legal guardian/next of kin, for the publication of any potentially identifiable images or data included in this article.

## Author Contributions

YL: resources and writing original draft preparation. LT: software, investigation, and visualization. YX: formal analysis and data curation. JL: writing review and editing, supervision, project administration, and funding acquisition. All authors have read and agreed to the published version of the manuscript.

## Funding

This work was supported by the Lanzhou Talent Innovation and Entrepreneurship Project (Grant Number: 2020-RC-49) and the Basic Research Innovation Group Project of Gansu Province (Grant Number: 21JR7RA432).

## Conflict of Interest

The authors declare that the research was conducted in the absence of any commercial or financial relationships that could be construed as a potential conflict of interest.

## Publisher's Note

All claims expressed in this article are solely those of the authors and do not necessarily represent those of their affiliated organizations, or those of the publisher, the editors and the reviewers. Any product that may be evaluated in this article, or claim that may be made by its manufacturer, is not guaranteed or endorsed by the publisher.
