# Supplementary material for: Giant Hepatic Hemolymphangioma With Peritoneal Effusion in Children: A Case Report and Literature Review
Source: Front Pediatr. 2022 Feb 18;10:817521. doi: 10.3389/fped.2022.817521 (PMC8894606; doi:10.3389/fped.2022.817521)
Supplement: Supplementary file 1 [file Data_Sheet_1.docx]

Supplementary Material

**
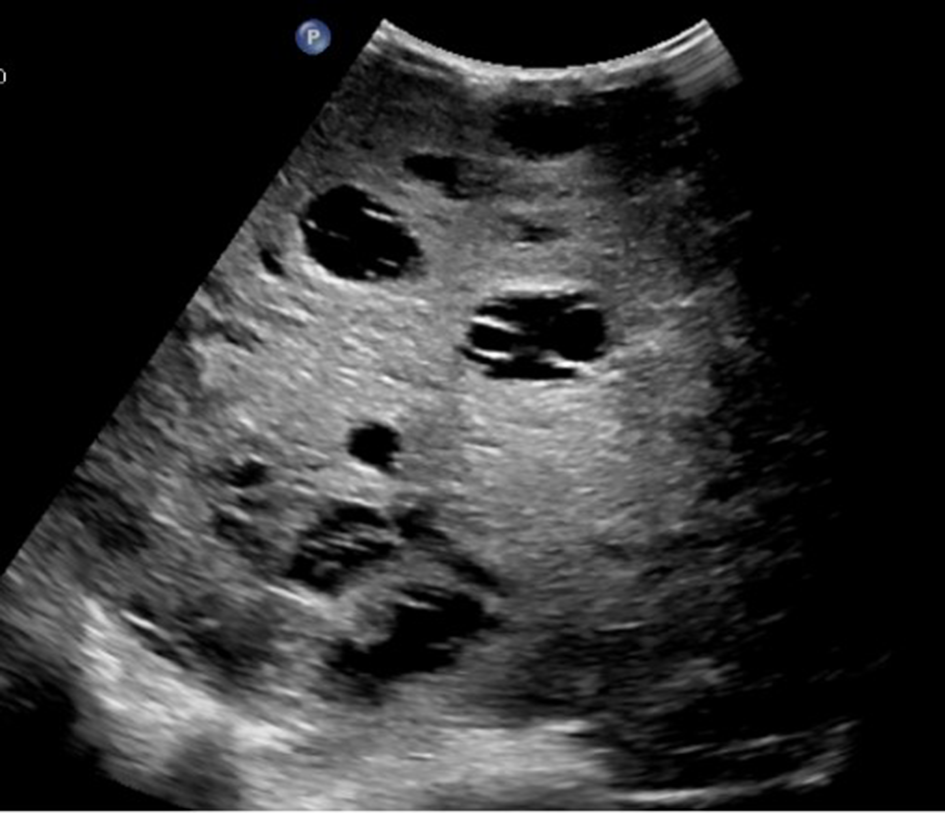
**

**Supplementary Figure 1.** Preoperative ultrasound of a giant solid-cystic occupancy in the right lower abdomen with mixed signal.

**
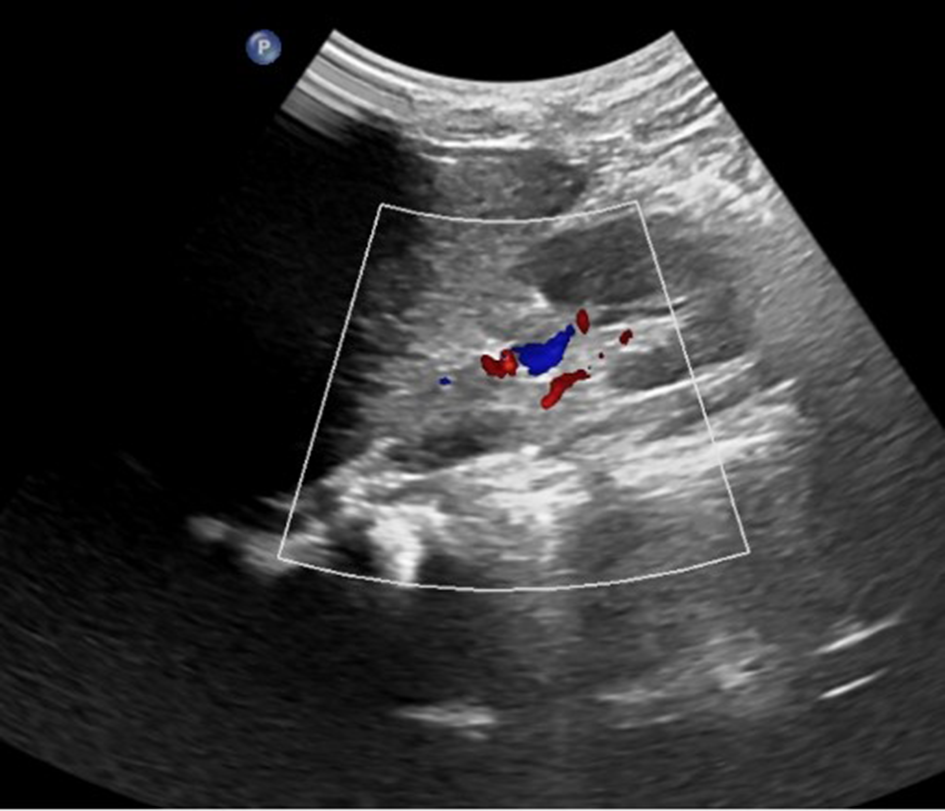
**

**Supplementary Figure 2.** Ultrasound review at 3 weeks postoperatively showed good recovery of the operated area.
